# The Psychological Experience of COVID-19 Vaccination and Its Impact on the Willingness to Receive Booster Vaccines among the Chinese Population: Evidence from a National Cross-Sectional Study

**DOI:** 10.3390/ijerph19095464

**Published:** 2022-04-29

**Authors:** Yudong Miao, Yi Li, Wanliang Zhang, Jian Wu, Jianqin Gu, Meiyun Wang, Wei Wei, Beizhu Ye, Chengyuan Miao, Clifford Silver Tarimo, Wenyong Dong

**Affiliations:** 1 Department of Health Management, College of Public Health, Zhengzhou University, Zhengzhou 450001, China; meldon@zzu.edu.cn (Y.M.); ly07263026@163.com (Y.L.); reynolds2475747505@163.com (W.Z.); jianwuzzu@163.com (J.W.); yebeizhu@163.com (B.Y.); mcy15518010986@163.com (C.M.); cliffordtarimo94@gmail.com (C.S.T.); 2 Henan Research Center for HTA, Zhengzhou 450001, China; 3 Research Center for Lifestyle Medicine, School of Medicine, Southern University of Science and Technology, Shenzhen 518055, China; jiangpanzy@sina.com; 4 Department of Hypertension, Henan Provincial People’s Hospital, People’s Hospital of Zhengzhou University, Zhengzhou 450003, China; mywang@ha.edu.cn (M.W.); wwei@ha.edu.cn (W.W.)

**Keywords:** COVID-19, COVID-19 vaccination, psychological experience, willingness, booster vaccines

## Abstract

The purpose of this study was to assess the psychological experience of COVID-19 basic vaccination, the willingness to receive booster vaccines, and to determine their relationships among Chinese people. Between 6 August 2021 and 9 August 2021, a research firm performed a national cross-sectional online survey among Chinese individuals (aged over 18), using the snowball sampling approach, with 26,755 participants. Factor analysis and binary logistic regression were used to evaluate the existing associations. The overall COVID-19 vaccination psychological experience score of the participants was 25.83 (25.78~25.89; scores ranged from 7–35). A total of 93.83% (95%CI = 93.54~94.12) of respondents indicated a willingness to receive booster vaccines. After classifying psychological experiences associated with COVID-19 vaccination into positive and negative experiences and adjusting for confounding factors, for the former, the willingness to receive booster vaccines for participants with the highest scores of 13–15 was 3.933 times higher (OR = 3.933, 95%CI = 3.176~4.871) than participants who obtained scores of 3–9, and for the latter, the willingness to receive booster vaccines for participants with the highest scores of 19–20 was 8.871 times higher (OR = 8.871, 95%CI = 6.240~12.612) than participants who obtained scores of 4–13. Our study suggests that a good psychological experience with vaccination is positively associated with an increased willingness to receive booster vaccines.

## 1. Introduction

Vaccination is a critical preventive measure for containing the COVID-19 pandemic [1]. However, studies have shown that a rejection rate of more than 10% reduces the population benefits of the COVID-19 vaccine [2]. Thus, only wide vaccination coverage will effectively halt the spread of the COVID-19 epidemic. Adult-targeted vaccines such as the American Pfizer-BioNTech COVID-19 vaccine, the Chinese Sinovac COVID-19 vaccine, and the Russian “Sputnik V” COVID-19 vaccine have all been thoroughly tested and approved for widespread use [3].

Numerous preliminary studies have shown that willingness to receive a COVID-19 vaccine is considered the primary factor affecting its coverage [4,5]. Over the last decade, global vaccination willingness has generally decreased, and the problem is more acute in developed countries [6]. On average, more than 30,000 people die from vaccine-preventable diseases in the United States each year [7]. A survey conducted in Taizhou, China, showed that the majority of respondents (*n* = 1435 [91.1%]) were willing to receive a booster vaccination against COVID-19 [8]. According to a survey conducted in Poland, 71% of respondents indicated a willingness to receive a booster dose of COVID-19 [9].

The factors that influence a person’s willingness to vaccinate are numerous and complex. Reduced willingness to vaccinate is generally associated with decreased trust, fear of side effects, a lack of pertinent information, personal experience [10,11,12,13], decreased trust in the government [14,15], risk perception [16], and vaccination experience [17]. In addition, even the parent’s own vaccination experience was found to influence their attitude toward their children’s vaccination [18]. Concerns about the safety of the COVID-19 vaccine, a lack of trust in the vaccine’s research and related information, as well as a lack of trust in the government have all been linked to people’s unwillingness and hesitation to receive the vaccine [14,15,19].

However, there is still a dearth of empirical research into the psychological experience of COVID-19 vaccination and its impact on COVID-19 vaccination willingness. However, during the vaccination process, psychological experiences such as pain and anxiety are common phenomena [20]. According to a survey, approximately 15% of nursing staff have had some level of fear of vaccination [21]. In addition, a study in Italy revealed that 40.7% of participants felt slightly nervous when receiving the first dose, and 32.7% reported feeling slightly nervous when receiving the second dose; 26.4% reported feeling scared when receiving the first dose, and 21.8% reported feeling scared when receiving the second dose [22]. Therefore, the vaccination psychological experience and attitude toward the second and even the third round of vaccination need to be researched.

As of 17 January 2022, a total of 2942.111 million doses of COVID-19 vaccine had been reported by 31 Chinese provinces (autonomous regions and municipalities directly under the Central Government) and Xinjiang Production and Construction Corps [23]. At the moment, China is experiencing a spotty outbreak of the epidemic, and experts suggest that booster vaccination can be administered. Given the fact that the main objective of the vaccination program is to reach a large fraction of the population, we can assume that a better psychological experience will make a positive contribution to the rapid increase in the intensive vaccination rate.

Therefore, we conducted a nationwide survey to assess the psychological experience of COVID-19 basic vaccination, the willingness to receive booster vaccines, and to determine their relationships among Chinese people. This will not only aid in explaining the critical links in the enhancement of the COVID-19 vaccination service experience but will also assist China and the rest of the world in rapidly promoting effective decision-making regarding COVID-19 vaccine booster vaccination.

## 2. Materials and Methods

### 2.1. Procedures, Participants and Study Design

**Figure 1:**
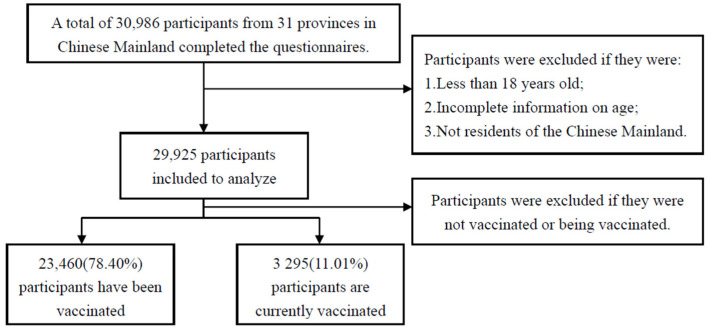
Inclusion and exclusion criteria.

We adopted the research procedures from a previously published study [24]. We conducted this study by snowball sampling. In this study, sample saturation occurred when the sample reached a size at which the vaccine hesitancy rate remained constant or did not change significantly as the snowball sample size increased. When the number of valid questionnaires reached 21,780, we found that the sample began to saturate (Figures S1 and S2). We concluded the online survey on 9 August 2021, when the total number of valid questionnaires reached 29,925. We included 23,460 respondents who had been vaccinated previously plus 3295 respondents who received a vaccine shortly prior to the execution of this study, making a total of 26,755 survey subjects (Figure 1). In this study, we converted the psychological experience of COVID-19 vaccination into an index that can be evaluated numerically and collected data about the population’s willingness to receive booster vaccines, and on this basis, judge the relationship between the psychological experience of COVID-19 vaccination and the willingness to receive booster vaccines.

### 2.2. Assessments

We collected various information, including sex, age, marital status, educational status, ethnic groups, religion, subjective social status, EQ-5D, chronic condition, smoking status, drinking status, washing hands status, wearing mask status, gathering activities status, adverse reactions, COVID-19 conspiracy beliefs, risk of COVID-19 infection, curability of COVID-19, channel of vaccine information, vaccine conspiracy beliefs, convenience of vaccination, trust in doctors, and trust in developers.

We reviewed previous articles on vaccine hesitation and then developed a questionnaire to assess psychological experience. Psychological experience was primarily composed of five-point Likert scale items. These included the following: Item 1. Do you feel humiliated about COVID-19 vaccination? Item 2. Do you feel sick about COVID-19 vaccination? Item 3. Do you feel happy about COVID-19 vaccination? Item 4. Do you feel angry about COVID-19 vaccination? Item 5. Do you feel relieved about COVID-19 vaccination? Item 6. Do you feel excited about COVID-19 vaccination? Item 7. Do you feel anxious about COVID-19 vaccination? We assign a score of 1 to 5 on the basis of the quality of the participants’ psychological experiences, with 5 points awarded for a good experience and 1 point awarded for a bad experience. For example, item 1 includes (1) very humiliated, (2) humiliated, (3) general, (4) not humiliated, and (5) not at all. We define option (1) as 1 point, option (2) as 2 points, option (3) as 3 points, option (4) as 4 points, and option (5) as 5 points. Item 3 includes (1) very happy, (2) happy, (3) general, (4) not happy, and (5) not at all. We define option (1) as 5 points, option (2) as 4 points, option (3) as 3 points, option (4) as 2 points, and option (5) as 1 point. For each participant, the score is the sum of the scores for each item, and the total score ranges from 5 to 35 points.

We designed an item to assess responses for willingness to receive booster vaccines, including (1) very willing, (2) willing, (3) fair, (4) unwilling, (5) very unwilling, and (6) don’t know. During the data analysis, options (1) and (2) were combined and named as “willing to vaccinate,” while options (3), (4), (5), and (6) were combined and named as “hesitate to vaccinate”.

### 2.3. Statistical Analysis

An independent samples *t*-test or Chi-square test was carried out to test differences in willingness to receive booster vaccines across groups. A rank-sum test was used to test differences in COVID-19 vaccination psychological experience scores across groups. Then, factor analysis was used to establish the model and KMO statistics and Bartlett’s spherical test were used to verify whether factor analysis was appropriate, while principal component analysis (PCA) was used to generate factor variables. The maximum variance rotation method was used to rotate the factor matrix. The score was not normal, so we used the quartile method to categorize scores into four levels, namely Q1, Q2, Q3, and Q4. The collinearity test was carried out to assess the association between independent variables using a variance inflation factor (VIF) < 3, and no collinearity was detected. Binary logistic regression analysis was used to determine the factors associated with the willingness to receive booster vaccines and the psychological experience associated with the COVID-19 vaccination. Data analyses were conducted using SAS 9.4 software. Differences were regarded as statistically significant if *p*-values were less than 0.05.

## 3. Results

### 3.1. COVID-19 Vaccination Psychological Experience Score Status

**Figure 2:**
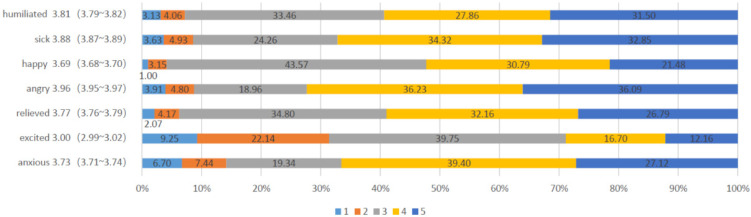
Percentage of participants’ scoring with each item. Note: The Cronbach’s Alpha = 0.709 and the KMO = 0.782.

A total of 26,755 participants were included in this study. A summary of sociodemographic information, lifestyle behavior, cognition, level of knowledge, and behavior toward COVID-19 and COVID-vaccine, plus many others, are detailed in Table 1. The average psychological experience with the COVID-19 vaccination service was 25.83 (95% CI: 25.78–25.89). Table 1 depicts the characteristics of participants with their respective levels of experience. Lower psychological experience of COVID-19 scores were observed with age between 18 and 29, men, below high school, minority, religious beliefs, not in marriage, suffered from chronic diseases, current smoker, current drinker, washing hands decreased, wearing mask decreased, gathering activities unchanged, adverse reactions, COVID-19 conspiracy beliefs, high risk of COVID-19 infection, medium possibility of curability of COVID-19, obtaining vaccine information through other ways, vaccine conspiracy beliefs, inconvenience of vaccination, and lower trust in doctors and developers. Each item is scored differently; item 4 has the highest score of 3.96 (95% CI: 3.95~3.97), and item 6 has the lowest score of 3.00 (95% CI: 2.99~3.02) (Figure 2).

### 3.2. Prevalence of Willingness to Receive Booster Vaccines

A summary of the participants’ sociodemographic information, lifestyle behavior, level of knowledge, willingness to receive booster vaccines, and others are provided in Table 1. In the total sample, 25,104 (93.83%, 95%CI: 93.54~94.12) participants expressed their willingness to uptake the booster vaccine. Lower willingness was observed among the population consisting of older (age ≥ 60 years), men, below high school, minority, religious beliefs, not in marriage, higher subjective social status, lower self-report health condition, suffered from chronic diseases, current smoker, current drinker, washing hands decreased, wearing mask decreased, gathering activities unchanged, COVID-19 vaccine adverse reactions, COVID-19 conspiracy beliefs, unsure the risk of COVID-19 infection, unsure the possibility of curability of COVID-19, obtaining vaccine information through other ways, vaccine conspiracy beliefs, inconvenience of vaccination, and lower trust in doctors and developers.

### 3.3. Associations between Psychological Experience and Willingness to Receive Booster Vaccines

**Figure 3:**
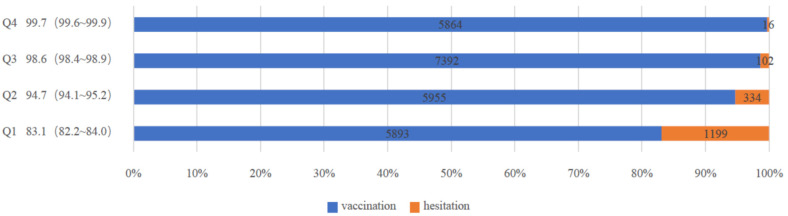
Number of hesitations and vaccinations per score interval. Note: We categorized the score of COVID-19 vaccination psychological experience by quartiles as Q1 (7–22 points), Q2 (23–25 points), Q3 (26–29 points) and Q4 (30–35 points).

**Figure 4:**
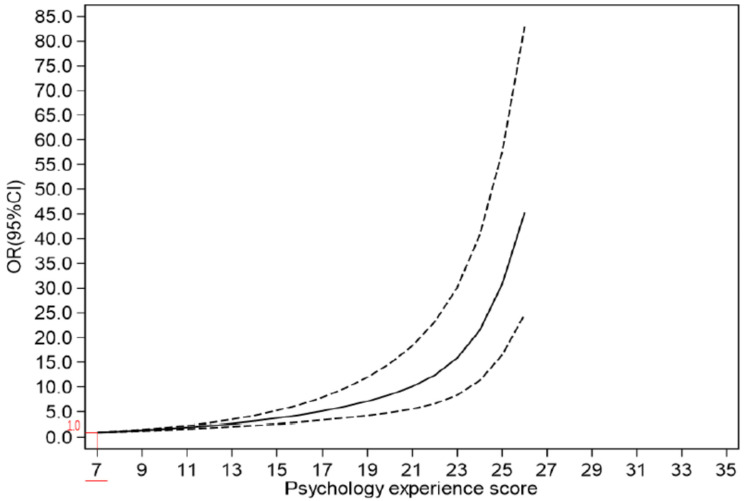
Correlation between willingness to uptake booster vaccines and psychological experience score. Note: The solid line represents the OR value, and the dashed line represents the 95% CI.

Participants in the Q1 category had the lowest willingness to receive booster vaccines at 83.1% (95% CI: 82.2~84.0), whereas participants in the Q4 category had the highest willingness to receive booster vaccines at 99.7% (95% CI: 99.6~99.9) (Figure 3). The higher the psychological experience score, the greater the willingness to receive booster vaccines. (Figure 4). After adjusting for confounding factors and dividing COVID-19 vaccination psychological experience into positive psychological experience and negative psychological experience (Table S1), for the former, participants in the Q2 category were 1.931 times (95%CI: 1.630~2.288) more willing to receive the booster dose than those in the Q1 category. Likewise, participants in the Q3 category were 2.460 times (95%CI: 2.111~2.867) more willing than those in the Q1 category, while those in Q4 were 3.933 times (95%CI, 3.176~4.871) more willing than those in Q1 category. In the latter, the willingness to receive booster vaccines among participants in the Q2 category was found to be 2.474 times higher (95%CI: 2.154~2.843) than for those in the Q1 category, while those in the Q3 category were 3.935 times (95%CI: 3.093~5.006) more likely than participants in the Q1 category. Participants in the Q4 category were 8.871 times (95%CI: 6.240~12.612) more likely than those in the Q1 category (Table 2).

## 4. Discussion

As found in this article, the average score of COVID-19 vaccination psychological experience among all participants was 25.83 (95%CI: 25.78~25.89), indicating a moderate level. These experiences are caused by a variety of factors, including fear of adverse effects as a result of prior experiences [10], fear of needles and injections [25], and others. According to a survey, approximately 5% of the difference in COVID-19 vaccine hesitation among adults may be explained by fear of injections; if the fear of blood injection injury is eliminated, slightly more than 10% of COVID-19 vaccine-hesitating patients may also be eliminated [25]. Another reason is a lack of trust in physicians and vaccine developers. Participants’ vaccination experience will be positive if they have a high level of trust in physicians and vaccine developers. When participants lack confidence in physicians and vaccine developers, their vaccination experience will be bad. In addition, when the participants consider receiving COVID-19 vaccines convenient, their vaccination experience will be better compared with when the services are inconvenient.

As shown in the table, among all participants, 25,104 (93.83%, 95%CI: 93.54~94.12) participants expressed their willingness to receive booster vaccines. The increased willingness to accept booster vaccines may be largely due to the fact that China has enacted a vaccine management law and approved the World Health Organization’s assessment of its National Vaccine Regulatory System (NRS), which builds public confidence in vaccines and ensures their quality and supply [5,26,27]. Secondly, China has continued to strengthen post-market surveillance of vaccines, focusing on vaccine efficacy and safety [28] and tracking vaccine-preventable disease incidence and public acceptance of vaccines on a continuous basis. In addition, the tracking of vaccine use experience and development of vaccine big data are still in progress. Thirdly, China has strengthened risk communication to educate recipients and the general public about the benefits and risks of vaccination and to spread the scientific concept that the overall benefits of vaccination far outweigh the risks. Finally, China has engaged in expanding vaccine availability, which requires vaccination services to be tailored to the characteristics of the jurisdiction area and population, as well as a reasonable distribution of vaccination clinics [24].

The results of this survey can prove that a positive correlation exists between a higher psychological experience score and a greater willingness to receive booster vaccines. As with previous research, the overall attitudes toward vaccination have an effect on vaccination [29], and a good vaccination experience increases residents’ willingness to receive booster vaccines. A survey on the willingness to be vaccinated conducted among nurses in Hong Kong showed a number of psychological factors that affect people’s willingness to be vaccinated [30,31]. A series of negative emotions such as anger, fear, disgust, anxiety, disgust, worry, etc. can affect people’s vaccination behavior [10]. Therefore, in order to increase the population’s willingness to vaccinate and to effectively promote the vaccination program, measures should be taken to improve the psychological experience of vaccination. These include, first and foremost, informing residents about the vaccine’s safety and effectiveness, as well as other pertinent information, and, in particular, providing strong evidence of vaccine safety and efficacy from field trials. Secondly, the authorities should disseminate vaccine-related information through community workers and some official channels so as to improve residents’ trust in information [32]. Vaccination points should be set up to improve the convenience of vaccination. Further, we can accomplish this goal by strengthening the population’s awareness of the risk of contracting COVID-19. Studies have shown that people from regions that are severely impacted by COVID-19 expressed a higher vaccination intention. Some studies have shown that watching nurses prepare for injections or watching other people receive injections will aggravate the anxiety of people waiting in line and affect the vaccination experience of people [33]. Authorities should create a welcoming vaccination environment to relieve the nervousness of the crowd waiting for vaccination.

This is the first survey of its kind in mainland China, encompassing 31 provinces and revealing the most recent status of Chinese residents’ vaccination and hesitation. This study, however, had some limitations. Due to the severity of the current epidemic, face-to-face offline surveys were not possible. As a result, the article employed the snowball sampling method of online surveys, which may have limited the representativeness of the sample. As a result, we used the method of increasing the sample size to avoid it, and the next study discovered that it required correction by a larger and more representative study. Furthermore, the effect of socioeconomic status on willingness to receive booster vaccines observed in this study may not be applicable to people who do not have access to the internet. Secondly, the survey and research method used in the article is a cross-sectional design. The analysis, similar to other cross-sectional studies, can only draw a correlation between specific factors but cannot make causal inferences. Since this research is an online survey, it is hoped that offline surveys of larger and more representative samples can be carried out to analyze in detail how the vaccination experience affects vaccination willingness.

## 5. Conclusions

In conclusion, a positive correlation exists between a higher psychological experience score and a greater willingness to receive booster vaccines. Therefore, when implementing a nationwide vaccination program, whether it is the COVID-19 vaccine or other vaccines, we should focus on residents’ psychological experience to enhance the acceptance of vaccines.

## Figures and Tables

**Table 1:** Sociodemographic information, cognition of COVID-19 pandemic, COVID-19 vaccine exception, trust in healthcare system, COVID-19 vaccination psychological experience score, and the willingness to uptake booster vaccines among all study participants.

Covariates	Total (%)	*p*-Value ^a^	COVID-19 Vaccination Psychological Experience Score (Mean, 95% CI)	*p*-Value ^a^	The Willingness to Uptake Booster Vaccines (95% CI)	*p*-Value ^a^
Total participants	26,755 (100)		25.83 (25.78~25.89)		93.83 (93.54~94.12) ^b^	
Demographic characteristics						
Age (years)		<0.001		<0.001		<0.001
18–29	11,694 (43.7)		25.22 (25.14~25.29)		91.59 (91.08~92.09) ^b^	
30–39	10,735 (40.1)		26.29 (26.21~26.37)		95.61 (95.22~96.00) ^b^	
40–49	3044 (11.4)		26.49 (26.34~26.63)		96.16 (95.47~96.84) ^b^	
50–59	1036 (3.9)		26.20 (25.97~26.44)		94.40 (93.00~95.80) ^b^	
60–	246 (0.9)		25.71 (25.18~26.24)		91.46 (87.95~94.98) ^b^	
Sex		<0.001		<0.001		<0.001
Men	12,685 (47.4)		25.59 (25.51~25.66)		92.52 (92.06~92.98) ^b^	
Women	14,070 (52.6)		26.06 (25.99~26.13)		95.01 (94.65~95.37) ^b^	
Educational status		<0.001		<0.001		<0.001
Below high school	2948 (11.0)		24.61 (24.45~24.78)		87.65 (86.46~88.84) ^b^	
High school graduate	7032 (26.3)		26.11 (26.00~26.21)		95.39 (94.90~95.88) ^b^	
University graduate	16,775 (62.7)		25.93 (25.87~26.00)		94.26 (93.91~94.61) ^b^	
Ethnic groups		<0.001		<0.001		<0.001
Han	25,741 (96.2)		25.88 (25.82~25.93)		94.12 (93.83~94.41) ^b^	
Minority	1014 (3.8)		24.78 (24.50~25.07)		86.39 (84.28~88.50) ^b^	
Religion		<0.001		<0.001		<0.001
Atheist	23,228 (86.8)		25.97 (25.92~26.03)		94.45 (94.15~94.74) ^b^	
Others	3527 (13.2)		24.90 (24.75~25.06)		89.76 (88.76~90.77) ^b^	
Marital status		<0.001		<0.001		<0.001
Married	16,666 (62.3)		26.37 (26.30~26.43)		95.49 (95.18~95.81) ^b^	
Others	10,089 (37.7)		24.95 (24.87~25.04)		91.08 (90.52~91.64) ^b^	
Subjective social status						
Society level	7.64 ± 2.092	<0.001			7.66 ± 2.09 ^c^	<0.001
Community level	7.98 ± 2.13	<0.001			8.00 ± 2.12 ^c^	<0.001
EQ-5D	85.26 ± 13.64	<0.001			85.71 ± 13.26 ^c^	<0.001
Chronic condition		<0.001		<0.001		<0.001
Yes	3251 (12.2)		24.39 (24.23~24.54)		87.63 (86.50~88.77) ^b^	
No	23,504 (87.8)		26.03 (25.98~26.09)		94.69 (94.40~94.97) ^b^	
Smoking status		<0.001		<0.001		<0.001
Current smoker	7763 (29.0)		24.28 (24.18~24.38)		89.63 (88.95~90.31) ^b^	
Former smoker	1482 (5.5)		26.70 (26.47~26.92)		94.33 (93.15~95.51) ^b^	
Never smoker	17,510 (65.4)		26.70 (26.47~26.92)		95.65 (95.35~95.95) ^b^	
Drinking status		<0.001		<0.001		<0.001
Current drinker	16,122 (60.3)		25.25 (25.19~25.32)		92.28 (91.87~92.70) ^b^	
Former drinker	930 (3.5)		26.38 (26.09~26.67)		94.19 (92.69~95.70) ^b^	
Never drinker	9703 (36.3)		26.74 (26.66~26.83)		96.36 (95.99~96.73) ^b^	
Health behaviors						
Washing hands		<0.001		<0.001		<0.001
Increased	23,491 (87.8)		26.14 (26.09~26.20)		95.26 (94.99~95.53) ^b^	
Unchanged	2919 (10.9)		23.70 (23.56~23.83)		84.14 (82.81~85.46) ^b^	
Decreased	345 (1.3)		22.84 (22.43~23.26)		78.55 (74.20~82.90) ^b^	
Wearing mask		<0.001		<0.001		<0.001
Increased	25,051 (93.6)		26.05 (25.99~26.10)		94.99 (94.72~95.26) ^b^	
Unchanged	1323 (4.9)		22.71 (22.50~22.92)		77.40 (75.14~79.66) ^b^	
Decreased	381 (1.4)		22.64 (22.24~23.04)		74.28 (69.87~78.69) ^b^	
Gathering activities		<0.001		<0.001		<0.001
Increased	7116 (26.6)		25.01 (24.90~25.12)		92.45 (91.84~93.07) ^b^	
Unchanged	2428 (9.1)		23.44 (23.29~23.59)		83.28 (81.79~84.76) ^b^	
Decreased	17,211 (64.3)		26.51 (26.45~26.57)		95.89 (95.59~96.18) ^b^	
COVID-19 conspiracy beliefs		<0.001		<0.001		<0.001
Level 1	2393 (8.9)		25.61 (25.45~25.77)		93.44 (92.45~94.43) ^b^	
Level 2	10,479 (39.2)		27.34 (27.26~27.42)		97.21 (96.90~97.53) ^b^	
Level 3	6812 (25.5)		25.28 (25.19~25.38)		93.35 (92.76~93.94) ^b^	
Level 4	7071 (26.4)		24.21 (24.10~24.31)		89.41 (88.69~90.12) ^b^	
Risk of COVID-19 infection		<0.001		<0.001		<0.001
Very high	1886 (7.0)		24.10 (23.87~24.32)		93.48 (92.36~94.59) ^b^	
High	1895 (7.1)		23.57 (23.37~23.77)		86.12 (84.56~87.68) ^b^	
Medium	3889 (14.5)		24.83 (24.70~24.96)		90.69 (89.78~91.61) ^b^	
Low	14,080 (52.6)		26.32 (26.26~26.39)		95.62 (95.28~95.96) ^b^	
No	4195 (15.7)		27.23 (27.09~27.36)		96.09 (95.50~96.68) ^b^	
Not sure	810 (3.0)		24.25 (23.99~24.51)		84.94 (82.47~87.41) ^b^	
Curability of COVID-19		<0.001		<0.001		<0.001
Very high	11,892 (44.4)		27.03 (26.95~27.11)		96.84 (96.52~97.15) ^b^	
High	9811 (36.7)		25.17 (25.09~25.24)		93.48 (92.99~93.97) ^b^	
Medium	2803 (10.5)		23.74 (23.59~23.88)		86.73 (85.47~87.99) ^b^	
Low	1205 (4.5)		25.08 (24.84~25.32)		89.46 (87.72~91.20) ^b^	
No	427 (1.6)		26.62 (26.14~27.10)		93.68 (91.36~95.99) ^b^	
Not sure	617 (2.3)		23.93 (23.64~24.22)		82.33 (79.32~85.35) ^b^	
Vaccine adverse reactions		<0.001		<0.001		<0.001
Yes	5079 (19.0)		23.61 (23.49~23.73)		91.06 (90.28~91.85) ^b^	
No/Unclear	21,676 (81.0)		26.35 (26.30~26.41)		94.48 (94.17~94.78) ^b^	
Channel of vaccine information		<0.001				<0.001
Community worker	7872 (29.4)		26.55 (26.45~26.66)	<0.001	96.00 (95.57~96.43) ^b^	
Internet	13,921 (52.0)		25.67 (25.60~25.74)		93.98 (93.59~94.38) ^b^	
Others	4962 (18.5)		25.15 (25.03~25.27)		89.96 (89.13~90.80) ^b^	
Vaccine conspiracy beliefs		<0.001		<0.001		<0.001
Level 1	1874 (7.0)		25.00 (24.82~25.18)		91.78 (90.54~93.03) ^b^	
Level 2	11,357 (42.4)		27.97 (27.90~28.05)		98.00 (97.74~98.26) ^b^	
Level 3	6265 (23.4)		25.43 (25.35~25.51)		96.15 (95.68~96.63) ^b^	
Level 4	7259 (27.1)		23.05 (22.97~23.14)		85.82 (85.02~86.63) ^b^	
Convenience of vaccination		<0.001		<0.001		<0.001
High	25,5559 (95.5)		25.97 (25.92~26.02)		94.50 (94.22~94.78) ^b^	
Medium	969 (3.6)		22.96 (22.75~23.18)		79.67 (77.13~82.21) ^b^	
Low	227 (0.8)		22.85 (22.33~23.37)		78.85 (73.50~84.21) ^b^	
Trust in doctors		<0.001		<0.001		<0.001
Level 1	7033 (26.3)		23.16 (23.07~23.25)		85.58 (84.76~86.40) ^b^	
Level 2	7979 (29.8)		25.04 (24.96~25.12)		94.30 (93.79~94.81) ^b^	
Level 3	5159 (19.3)		27.15 (27.05~27.26)		97.81 (97.41~98.21) ^b^	
Level 4	6584 (24.6)		28.61 (28.52~28.71)		98.95 (98.71~99.20) ^b^	
Trust in developers		<0.001		<0.001		<0.001
Level 1	7580 (28.3)		23.23 (23.14~23.32)		85.34 (84.55~86.14) ^b^	
Level 2	7930 (29.6)		25.21 (25.13~25.29)		95.44 (94.98~95.89) ^b^	
Level 3	11,245 (42.0)		28.03 (27.95~28.10)		98.42 (98.19~98.65) ^b^	

CI, confidence interval. We categorized the score of COVID-19 conspiracy beliefs by quartiles as level 1 (7–21 points), level 2 (22–28 points), level 3 (29–35 points), and level 4 (36–42 points), and the score of vaccine conspiracy beliefs by quartiles as level 1 (7–25 points), level 2 (26–29 points), level 3 (30–35 points), and level 4 (36–42 points). We categorized the score of trust in doctors by quartiles as level 1 (9–30 points), level 2 (31–36 points), level 3 (37–40 points), and level 4 (41–45 points), and the score of trust in developers by quartiles as level 1 (5–18 points), level 2 (19–21 points), and level 3 (22–25 points). ^a^ Difference between categories within each variable. ^b^ Row percentages derived from the total number in the corresponding row. ^c^ Mean ± standard deviation for variables. Student’s *t*-tests for continuous variables and Chi-square tests for categorical variables.

**Table 2:** Relationship between the COVID-19 vaccination psychological experience score of participants and the willingness to uptake booster vaccines.

Model		Number of Surveys	Tendency to Hesitate Rate (%, 95%CI)	Unadjusted Variable		Adjusted Variable	
				OR	95%CI	OR	95%CI
Negative psychological experience model	Q1	7169	16.1 (15.3~17.0)	1.000		1.000	
	Q2	9060	4.1 (3.7~4.5)	4.495	3.981~5.075	2.474	2.154~2.843
	Q3	4560	1.9 (1.5~2.3)	10.012	8.015~12.506	3.935	3.093~5.006
	Q4	5966	0.6 (0.4~0.8)	31.701	22.708~44.255	8.871	6.240~12.612
Positive psychological experience model	Q1	1,0750	9.6 (9.0~10.1)	1.000		1.000	
	Q2	3822	5.3 (4.6~6.1)	1.885	1.615~2.201	1.931	1.630~2.288
	Q3	6919	4.2 (3.7~4.6)	2.439	2.133~2.789	2.460	2.111~2.867
	Q4	5264	2.4 (2.0~2.8)	4.371	3.619~5.278	3.933	3.176~4.871

We categorized the score of COVID-19 vaccination negative psychological experience by quartiles as Q1 (4–13 points), Q2 (14–16 points), Q3 (17–18 points), and Q4 (19–20 points), and the score of COVID-19 vaccination positive psychological experience by quartiles as Q1 (3–9 points), Q2 (10–10 points), Q3 (11–12 points), and Q4 (13–15 points).We adjusted age, sex, educational status, ethnic groups, religion, marital status, subjective social status in China, subjective social status in one’s community, body mass index, chronic condition, smoking status, drinking status, health behaviors, COVID-19 conspiracy beliefs, risk of COVID-19 infection, curability of COVID-19, channel of vaccine information, vaccine conspiracy beliefs, trust in doctors, trust in developers, and convenient vaccination.CI, confidence interval. OR, odds ratios.

## Data Availability

All data can be acquired by contacting the corresponding author.

## Supplementary Materials

The following supporting information can be downloaded at: https://www.mdpi.com/article/10.3390/ijerph19095464/s1, Table S1: Factor loading matrix of Chinese residents’ vaccination psychological experience model; Figure S1: Sample saturation, Figure S2: Sample Size Calculation Formula.
