# Supplementary material for: The Psychological Experience of COVID-19 Vaccination and Its Impact on the Willingness to Receive Booster Vaccines among the Chinese Population: Evidence from a National Cross-Sectional Study
Source: Int J Environ Res Public Health. 2022 Apr 29;19(9):5464. doi: 10.3390/ijerph19095464 (PMC9100074; doi:10.3390/ijerph19095464)
Supplement: Supplementary file 1 [file ijerph-19-05464-s001.zip › ijerph-1658444-supplementary.pdf]

**Table S1.** Factor loading matrix of Chinese residents' vaccination psychological experience model.

| Items | Negative Psychological Experience Model | | Positive Psychological Experience Model | |
|---|---|---|---|---|
| | **Classification** | **Load** | **Classification** | **Load** |
| | Anxious | 0.831 | Excited | 0.744 |
| | Angry | 0.892 | Relieved | 0.839 |
| | Sick | 0.892 | Happy | 0.825 |
| | Humiliated | 0.760 | | |
| Eigenvalues | | 3.006 | | 2.029 |
| Variance contribution rate (%) | | 42.942 | | 28.988 |
| Cumulative variance contribution rate (%) | | 42.942 | | 71.930 |

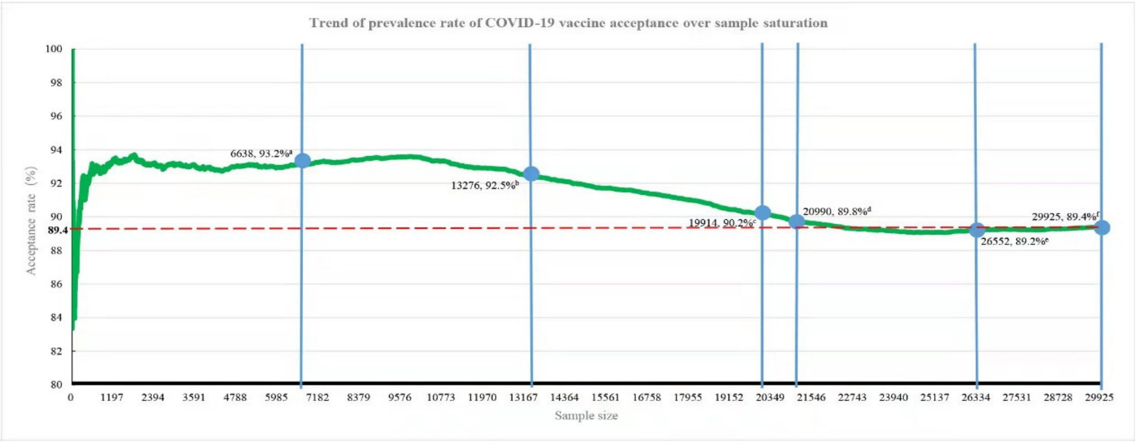


**Figure S1.** Sample saturation.

$$n = \frac{Z^2_{\alpha/2} * P * (1 - P)}{d^2}$$

**Figure S2.** Sample Size Calculation Formula.

Note: The minimum sample size based on the COVID-19 vaccination coverage rate of 83.43% in the preliminary online survey, an allowable error of 1% and consider the missing 20% sample size.
